# Imaging the Renal Microcirculation in Cell Therapy

**DOI:** 10.3390/cells10051087

**Published:** 2021-05-02

**Authors:** Katerina Apelt, Roel Bijkerk, Franck Lebrin, Ton J. Rabelink

**Affiliations:** 1 Department of Internal Medicine-Nephrology, Leiden University Medical Center, 2333ZA Leiden, The Netherlands; k.apelt.nier@lumc.nl (K.A.); r.bijkerk@lumc.nl (R.B.); f.lebrin@lumc.nl (F.L.); 2 Einthoven Laboratory of Vascular and Regenerative Medicine, Leiden University Medical Center, 2333ZA Leiden, The Netherlands; 3 Physics for Medicine Paris, Inserm, CNRS, ESPCI Paris, Paris Sciences et Lettres University, 75005 Paris, France

**Keywords:** kidney, microcirculation, microvascular rarefaction, cell therapy, imaging

## Abstract

Renal microvascular rarefaction plays a pivotal role in progressive kidney disease. Therefore, modalities to visualize the microcirculation of the kidney will increase our understanding of disease mechanisms and consequently may provide new approaches for evaluating cell-based therapy. At the moment, however, clinical practice is lacking non-invasive, safe, and efficient imaging modalities to monitor renal microvascular changes over time in patients suffering from renal disease. To emphasize the importance, we summarize current knowledge of the renal microcirculation and discussed the involvement in progressive kidney disease. Moreover, an overview of available imaging techniques to uncover renal microvascular morphology, function, and behavior is presented with the associated benefits and limitations. Ultimately, the necessity to assess and investigate renal disease based on in vivo readouts with a resolution up to capillary level may provide a paradigm shift for diagnosis and therapy in the field of nephrology.

## 1. Introduction

The renal vasculature has an anatomically complex architecture, which reflects its unique physiological function [1]. Despite the highly dynamic adaptation of the microvascular network to hemodynamic changes, vascular dysfunction may be the consequence or even the cause of kidney disease development and progression [2,3]. Accordingly, microvascular dysfunction may serve as an early hallmark of fibrotic kidney injury, implicating that non-invasive assessment and validation of renal microvascular architecture and function would be a great improvement to assess efficacy of therapies aimed at reduction of renal fibrosis.

In clinical practice, severity of chronic kidney disease (CKD) is classified based on the glomerular filtration rate (GFR) which reflects mildly (60–89 mL/min/1.73 m${}^{2}$), moderately (30–59 mL/min/1.73 m${}^{2}$) or severely (15–29 mL/min/1.73 m${}^{2}$) decreased kidney function based on predefined categories [4]. Disease progression into advanced CKD and end-stage renal disease (ESRD) is defined by the occurrence of glomerulosclerosis and tubulointerstitial fibrosis with a GFR of less than 15 mL/min/1.73 m${}^{2}$. Several studies have shown that the phenomenon of loss of renal capillaries, i.e., renal microvascular rarefaction, closely correlates with kidney disease severity and is involved in the biology of subsequent progression towards CKD [3,5,6,7]. The formation of renal fibrosis was highlighted as a central characteristic for such peritubular capillary loss and ensuing renal epithelial loss. If the microvasculature of the kidney can be protected or even restored, renal structural tissue integrity would improve and disease progression may be prevented [2,8]. Additionally, a therapeutic window can potentially be defined to assess the severity of injury before the degree of renal damage is irreversible. In particular, utilization of cell therapies such as promising applications of mesenchymal stromal cells (MSCs) can be assessed and refined for treating renal vascular diseases. However, a detailed understanding of renal microvascular rarefaction is still hampered by the absence of imaging modalities that enable high-resolution and non-invasive monitoring of vessel architecture and functionality.

In this review, we summarize current knowledge of the renal microvasculature and the pathological mechanisms that can affect the microcirculation. We discuss the importance of pericytes and provide insights into their central role in causing renal disease progression through microvascular dysfunction and rarefaction, as well as the development of kidney fibrosis. We subsequently describe new developments in imaging techniques that could monitor such changes in the renal microcirculation.

## 2. The Renal Vasculature

### 2.1. The Renal Blood Circulation

The microvasculature of the human body consists of arterioles, capillaries, and venules and effectuates the exchange of oxygen, nutrients, and metabolites between the blood and the surrounding tissue [8,9]. Evidently, the vital function of the microcirculation is tightly regulated based on the organ’s metabolic need. The main responsibility of arterioles is to regulate the blood flow by adjusting the resistance to ensure that the vital exchange at the level of capillaries can be executed [10]. In this regard, continuous adaption to the homeostatic demand of the underlying tissue is mainly dependent on the dynamic plasticity of endothelial cells. At the same time, endothelial health of the microcirculation is relying on the close intercellular communication with pericytes that physically stabilize the blood vessels, regulate angiogenesis, and control the blood flow [11,12].

**Figure 1:**
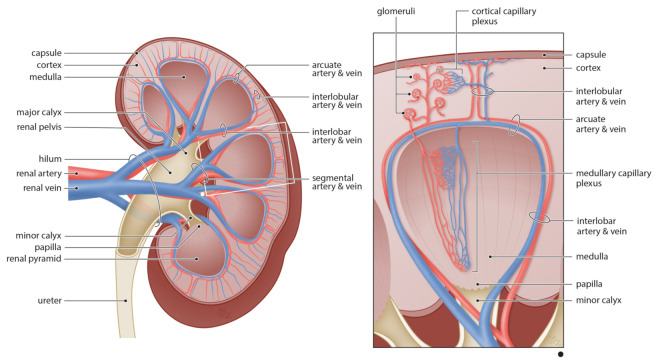
Anatomy of the renal vasculature. Blood enters the kidney via the renal artery which divides dichotomously into segmental arteries and branch progressively into interlobar arteries. Arcuate arteries, separating the border between the cortex and medulla, giving rise to interlobular arteries which further diverge to supply the glomeruli. Besides the glomerular capillary network, the renal microcirculation can be divided into cortical and medullary capillary plexus based on the anatomical location. Finally, blood flows via the arcuate, interlobar, and segmental veins to exit the kidney via the renal vein.

The kidney is a highly vascularized organ exhibiting unique morphological and functional characteristics which reflect the remarkable heterogeneity of its vascular network [1,10]. Blood enters the kidney via the renal artery through the hilum (Figure 1), which further divides dichotomously in the renal pelvis into segmental arteries and branch progressively at the level of the minor calyx into interlobar arteries which spread between the renal pyramids [1,13]. At the border between the cortex and the medulla, interlobar arteries flow into the arcuate arteries, forming an anatomical separation between both renal compartments [1]. In the cortex, interlobular arteries, also known as cortical radiate arteries or cortical penetrating arterioles, arise perpendicularly from arcuate arteries and diverge into afferent arterioles to supply the various branches of the glomerular tree. Depending on the location of the glomeruli, filtered blood come together in the cortical capillary plexus surrounding the proximal and distal tubules or the medullary capillary plexus at the level of the loop of Henle. Finally, blood drains into the venous system which runs in parallel to the arterial network exiting the kidney via the interlobular, arcuate, interlobar, segmental, and eventually the renal vein right above the ureter. Generally speaking, this basic vascular pattern is preserved across mammals [14,15].

### 2.2. The Capillary Networks of the Kidney

**Figure 2:**
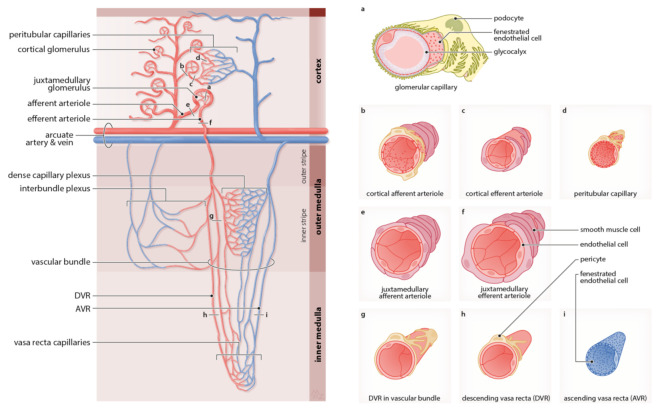
The renal microvascular network exhibits remarkable heterogeneity on morphological and functional level. The (**a**) glomerular capillaries have a fenestrated endothelium and are wrapped by podocytes. The highly muscularized (**b**) cortical afferent arteriole is surrounded by pericytes, in contrast to (**c**) cortical efferent arteriole which contains less smooth muscle cells and flows into the (**d**) peritubular capillaries that are highly surrounded by pericytes. The glomeruli situated close to the arcuate artery and vein are bigger in size, which is reflected by a larger vessel diameter of (**e**) juxtamedullary afferent arteriole and (**f**) juxtamedullary efferent arteriole. The vascular network of the medulla is supplied by efferent arterioles, which arise from the juxtamedullary glomeruli forming a dense capillary plexus and interbundle plexus in the inner stripe of the outer medulla. The (**g**) vascular bundle is wrapped by many pericytes and enter deeper into the inner medulla to form the vasa recta capillaries between the (**h**) descending vasa recta (DVR) and the fenestrated (**i**) ascending vasa recta (AVR).

The complexity of the renal microvasculature architecture is mirrored by a diverse morphology of the different renal blood vessels (Figure 2). The structural and functional heterogeneity of the renal endothelium and their surrounding perivascular cells go closely hand in hand with the type of capillary network [15,16,17]. In fact, the presence of various capillary beds is a remarkable feature ensuring filtration through the glomerular capillary network, as well as secretion and reabsorption via the peritubular capillary network and the medullary capillary network [1,13,14]. The cortical microcirculation mainly ensures the reabsorption of the glomerular filtrate, whereas salt and water excretion are predominantly regulated by the medullary microvascular compartment [14]. Interestingly, even though that the medulla makes approximately 30% of the total renal tissue mass, only 10% of the total renal blood flow (RBF) comprises this part [10]. Based on the anatomical position, the renal microcirculation can be divided into: (i) the cortical microcirculation; and (ii) the medullary microcirculation [13,14].

The cortical microcirculation is physically separated by the arcuate arteries that give rise to interlobular arteries that further branch from both sides into several afferent arterioles to supply the glomerular capillary network [14]. The branching occurs at a different angle depending on the location of the glomeruli within the cortex. Via the afferent arteriole, the glomerular capillary network consisting of 6–8 capillary loops is supplied with blood that exits via the efferent arteriole after being filtered [10]. Glomerular capillaries (Figure 2a) are formed of a thin, continuous, and mostly flat fenestrated endothelium which is covered by podocytes. The fenestrated areas can take up to 20–50% of their entire cell surface [16]. The cortical glomeruli compose 90% of all glomeruli present in the kidney, and therefore it is not surprising that most of the RBF predominantly flows through [13]. The remaining 10% of all glomeruli are situated at the cortico-medullary border and are bigger in size. Besides the size differences of the glomeruli, structural differences of afferent and efferent arterioles supplying the cortical and juxtamedullary glomeruli can be explained by the significance to preserve capillary pressure.

To ensure proper blood filtration, the diameter difference between cortical afferent (Figure 2b) and cortical efferent (Figure 2c) arterioles are 15 μm versus 10 μm [13]. Blood pressure is regulated at the side of afferent arterioles through the means of resistance changes explaining the continuous endothelium that is wrapped by smooth muscle cells (SMCs) [18]. A closer inspection of the afferent arteriole reveals the presence of two vascular segments rather than a uniform endothelium along the entire vessel length as commonly encountered. The proximal part of the afferent arteriole is composed of a non-permeable endothelium with tightly arrangement SMCs, which are required for vessel contraction. In contrast to the distal part, which is closely located to the glomerulus and consists of a permeable endothelium due to presence of fenestration. Interestingly, this fenestration is rather an uncommon feature in vessels with a high intravascular pressure. Besides, cube-shaped renin-producing pericytes wrapping the distal part of the afferent arteriole to mediate the regulation of local blood pressure in the glomerulus [17,19].

Filtered blood exits each of the cortical glomeruli via the efferent arteriole to come together in the dense cortical capillary plexus surrounding the renal tubules [10]. Apart from the glomerular capillary system, this second capillary compartment is known as the peritubular capillary system [16]. The peritubular capillaries (Figure 2d) are fenestrated and thin-walled with an average diameter of approximately 7 μm [13,15]. Those capillaries provide oxygen and nutrients to the tubules situated in the renal cortex, but not necessarily to the one they originate from [10,15]. Peritubular capillaries exhibit a more continuous endothelium, as well as a smaller diameter in comparison to the medullary capillaries of the vasa recta [13,16]. In renal disease, glomerular injury will finally influence the downstream sequentially arranged peritubular capillaries, and thereby accelerate renal disease progression [10,20].

The juxtamedullary afferent arteriole (Figure 2e) has an approximate diameter of 20 μm, whereas the juxtamedullary efferent arteriole (Figure 2f) has a thicker internal diameter of 20–25 μm [13]. The efferent arteriole exiting the juxtamedullary glomerulus is highly surrounded by several SMCs. This noticeable difference in vessel diameter, as well an increased muscularization, is raising the debate about the involvement of this glomerulus type in ischemia. It appears that the renal vascular architecture is organized in a certain way to preserve the medulla from ischemic damage. There is the hypothesis that afferent and efferent arterioles of the juxtamedullary glomeruli may not be responsible for regulating the medullary blood flow (MBF), but this would also be in potential contradiction with their role to control the glomerular filtration rate [21]. Instead, it seems that the descending vasa recta (DVR) of the vascular bundles situated in the inner stripe of the outer medulla is responsible for controlling the MBF which may explain the high number of pericytes wrapping this vessel compartment. The DVR plays therefore a key role for long-term regulation of arterial pressure [13]. Efferent arterioles connected to the juxtamedullary glomeruli flow through the vascular bundles, which are situated in the inner stripe of the outer medulla turn into the DVR. The DVR gives rise to the medullary capillary network that is connected to the ascending vasa recta (AVR) [1].

The medullary microcirculation starts when the efferent arterioles originated from the juxtamedullary glomeruli enter deeper into the tissue to supply the remaining 30% of the renal tissue known as the medulla [13,14]. The medulla of one renal pyramid is anatomically divided into two parts: the outer medulla situated right under the cortex, followed by the inner medulla that spreads until the apex of the parenchyma, called papilla. In general, the number of medullary capillaries increases from the renal pyramid tip. Closer observation indicates that the outer medulla can be further subdivided into the outer strip and the highly vascularized inner strip containing the dense capillary plexus and the interbundle plexus [15]. The interbundle capillaries are characterized by a fenestrated endothelium and are linked to the arcuate vein [14,22].

Each juxtamedullary efferent arteriole splits into several bundles, known as the vascular bundles, to form the branches of the DVR (Figure 2g) that have a larger diameter compared to the peritubular capillaries of the cortex [15,23]. Several pericytes are attached to the endothelium of the DVR of the vascular bundles [17]. Interestingly, the number of pericytes wrapping the vessels decreases in DVR (Figure 2h) of the inner medulla [15,17]. This morphological arrangement of the DVR in the outer and inner medulla compartment reflects its dual function [10]. To elaborate, vasoconstriction of the DVR mostly appears in the proximal part, which is situated in the outer medulla [15]. In the distal part, i.e., the inner medulla, however, mainly electrolyte exchange is taking place. This anatomical difference directly implies a distinct subpopulation of pericytes in terms of morphological appearance and functional property [15,17].

Deep within the inner medulla, various branches of the DVR split into a complex capillary network before connecting to the significantly smaller AVR [10]. The endothelium of the DVR is continuous, in contrast to the endothelial cells of the AVR (Figure 2i), which are highly fenestrated. Eventually, all the blood is collected from the AVR, as well as the peritubular capillaries of the cortical capillary plexus into the venous system. Generally, renal veins display an extremely thin vascular wall and interlobular, arcuate, and interlobar veins are fenestrated and containing diaphragms. Surprisingly, interlobular veins display in general greater similarities with peritubular capillaries than with veins due to the thin and highly fenestrated epithelium.

The different renal microvascular segments and their elegant morphological appearance on cell level raise the suspicion that the existing complexity is translated into an even more complicated disease mechanism. Therefore, the following section focuses on the underlying processes involved in renal microvascular malfunctions.

## 3. Renal Microvascular Malfunctions

### 3.1. Endothelial Dysfunction

The renal endothelium is mostly quiescent under physiological conditions; however, in response to microenvironmental changes, i.e., shear stress, hypoxia, oxidative stress, or inflammation, endothelial cell activation occurs and angiogenic growth factors are produced [24]. Depending on the trigger, endothelial cell activation may induce a pro-inflammatory and pro-thrombotic phenotype to promote immune cell adhesion and infiltration for microthrombi formation. However, to maintain the vascular barrier for suitable vasoregulatory function and permeability for solute transport, it is essential to tightly regulate the metabolic state of quiescent, as well as activated endothelial cells.

Tissue integrity and organ function are mainly dependent on a suitable perfusion of the microvascular network [8]. Therefore, it is not surprising that endothelial cells exhibit a high plasticity to ensure dynamic adaptation to environmental changes by adjusting capillary number, morphological shape, and function [1,2,8]. A prolonged period of elevated blood pressure, however, causes irreversible changes in the microcirculation, giving rise to injured endothelial cells characterized by impaired adaptation properties. This disrupted homeostasis is reflected by a reduction of nitric oxide (NO), hypoxia-inducible factor-1α (HIF-1α), and vascular endothelial growth factor (VEGF), together with an increase of other factors such as angiostatin, endostatin, and thrombospondin [1]. Notably, the susceptibility to endothelial dysfunction in the kidney is diverse and is dependent on the endothelial cell type located within the different compartments of the renal microcirculation [16,24]. To elucidate the heterogeneous phenotypes of renal endothelial cells and their different response to microvascular changes, Dumas et al. [25] recently provided a high-resolution atlas of the renal endothelium by using single-cell RNA sequencing.

Endothelial dysfunction often co-occurs with acute and progressive decline of kidney function [24]. This malfunction causes an increased vascular resistance, which is accompanied by a reduction in RBF [26]. A prolonged period of vasoconstriction causes an inadequate tissue perfusion and activation of stress and growth factors leading to morphological changes [1]. Depending on the severity and especially the duration of any given insult, the RBF can be altered irreversibly by introducing structural changes in the microcirculation. Those morphological changes are caused by a process known as microvascular remodeling. Microvascular remodeling is defined as the response to functional changes of the microvasculature, which consequently may cause alteration of microvascular architecture on structural level in a last attempt to reach hemodynamic homeostasis [1,2,8]. Finally, endothelial dysfunction may result in a phenomenon called ‘no-reflow’, whereby the perfusion cannot be restored, eventually leading to tubular epithelial cell damage that results in acute kidney injury (AKI) [24].

It is of great importance to highlight that endothelial dysfunction is not only associated with kidney disease, but also actively drives disease progression [24]. Renal microvascular malfunction is reflected by endothelial dysfunction provoked by cell injury, which disturbs the close interaction of pericytes with the endothelial layer and hampers cellular communication.

### 3.2. Pericyte Involvement in Renal Malfunction

A central feature of CKD is the progressive loss of the peritubular capillary network, a process that is referred to as rarefaction [27]. Tubulointerstitial fibrosis, as well as damaged tubular epithelium is preceded by this capillary rarefaction in the kidney [27], while this microvascular rarefaction is directly correlated with the severity of fibrosis [28,29]. Moreover, the extent of rarefaction has been found to predict the degree of interstitial damage as well as changes in the glomerular filtration rate in CKD patients [28]. These findings suggest an early, rate-limiting role for microvascular destabilization/loss in the development of CKD and the pathogenesis of fibrosis [30]. Chronic endothelial cell activation by cardiovascular risk factors can inflict the loss of pericytes that play a critical role in the stabilization and proliferation of capillaries via interactions with endothelial cells [31]. Indeed, accumulating evidence pinpoints towards the importance of pericytes and their involvement in renal microvascular health [17].

Pericytes are perivascular mural cells with elongated processes covering the endothelium that are embedded within the basement membrane of capillaries [32]. They are cells of mesenchymal origin and arise from the Forkhead box D1 (FoxD1)+ stromal progenitor population, which also give rise to the other mural cells of the kidney vasculature including SMCs, resident fibroblasts, renin cells, and mesangial cells [33], while all endothelial cells of the kidney vasculature originate from stem cell leukemia (SCL)+ progenitors [34]. Pericytes are different from resident (perivascular) fibroblasts since they are embedded in the vascular basement membrane, but most studies in the kidney do not distinguish between pericytes and perivascular fibroblasts [31,35], likely due to a lack of specific markers. Markers that are commonly used to identify pericytes include platelet-derived growth factor receptor-β (PDGFRβ), chondroitin sulfate proteoglycan NG2, α-smooth muscle actin (αSMA), cluster of differentiation 73 (CD73), and PDGFRα, but these markers identify different (overlapping) subsets of pericytes localized to different anatomical regions, reflecting the heterogeneity of this cell population [17] and most likely also functional heterogeneity. Pericytes tightly regulate vascular development, stabilization, maturation, and remodeling [11] and control blood flow by vasoconstriction. Pericytes are functionally regulated by vasoconstrictive factors such as angiotensin II and adenosine triphosphate (ATP), as well as by vasodilatory factors such as NO and prostaglandins [17]. The maturation of blood vessels is dependent on the recruitment of perivascular cells to stabilize the vasculature and control blood pressure [12].

In the kidney, pericytes are wrapping the distal parts of the afferent arterioles of the cortical glomeruli and are mainly present at the peritubular capillaries and at the vasa recta [13,19]. In addition, mesangial cells are a (specialized) subset of renal pericytes that are important in maintaining structural support for glomerular capillaries and regulating glomerular hemodynamics. Furthermore, contractile juxtaglomerular pericytes located at the arterioles mediate local glomerular blood pressure and affect systemic blood pressure through renin secretion [19]. Interestingly, renin precursor cells that are derived from the stromal compartment are spatiotemporally linked with blood vessel development while arterial branch formation was shown to be preceded by the appearance of renin-expressing cells at the point of branching [33,36]. Moreover, using transgenic renin reporter zebrafish, it was shown that renin-expressing cells precede angiogenic sprouts [37]. In the adult mouse kidney, cells of renin origin are also observed in perivascular locations and co-stain with pericyte markers (PDGFRβ/NG2) [38], suggesting a possible important role for this subset in vascular maintenance.

### 3.3. Endothelial Cell-Pericyte Signaling Interactions

Pericytes interact with the endothelial cells through a multitude of reciprocal interactions that regulate the signaling pathways required for stabilization and angiogenic sprouting. Pericyte signal to the endothelium through secreted factors such as VEGF, PDGF, transforming growth factor-β (TGF-β), and angiopoietin-1 (Ang-1), as well as by direct endothelial-pericyte crosstalk [39]. Similarly, the endothelium signals to surrounding stromal cells using factors such as angiopoietin-2 (Ang-2) and PDGF. Ang-2 negatively interferes with Ang-1-mediated Tie-2 signaling, which results in disruption of pericyte–endothelial cell interaction and subsequent vessel destabilization and abnormal microvascular remodeling [40,41]. The critical importance of the interaction between perivascular stromal cells and endothelial cells in maintenance of the capillary network is also evidenced by mouse studies, demonstrating that, when investment of pericytes is hampered, the capillary network is destabilized and rarefaction occurs [42]. For example, hyperglycemia increases endothelial Ang-2 expression causing perivascular stromal cells to migrate away from capillaries [43]. Recent studies from our laboratory demonstrated that both in rats [44] and in human donor kidneys [45] ischemia-reperfusion injury lead to a rapid elevation of the Ang-2/Ang-1 balance, which associated with a loss of microvascular integrity. Moreover, in patients with diabetes, reversal of capillary health and decrease in Ang-2/Ang-1 ratio and soluble thrombomodulin (endothelial cell injury marker) was observed within 12 months after a simultaneous kidney-pancreas transplantation [46]. Next to the angiopoietin/Tie2 pathways [47], endothelial–pericyte crosstalk is regulated by TGF superfamily signaling [48], VEGF [49], and sphingosine-1-phosphate (S1P) signaling pathways [50].

### 3.4. Pericytes as Precursor of Myofibroblasts

Murine genetic lineage-tracing models have demonstrated that pericytes (and other perivascular cells) are the major source of α-SMA positive myofibroblasts in mouse models of renal fibrosis [51,52]. In fact, a recent elegant study involving single cell RNA-sequencing pinpointed three main sources of myofibroblasts in human kidneys: (i) NOTCH3+RGS5+PDGFRα− pericytes; (ii) MEG3+PDGFRα+ fibroblasts; and (iii) COLEC11+CXCL12+ fibroblasts [53]. During pericyte-to-myofibroblast differentiation, cell cycle changes were observed that are consistent with differentiation and expansion, and enriched pathways included canonical WNT and activator protein-1 (AP-1) signaling, as well as activating transcription factor 2 (ATF2), PDGFRα, integrin, extracellular matrix (ECM) receptor interaction, and TGF-β signaling pathways [53]. It has been previously shown that a small fraction of the PDGFR+ cell population consists of perivascular Gli1+ progenitors that mark a perivascular MSC-like cell population which were demonstrated to also be key contributors to injury-induced organ fibrosis via generating myofibroblasts [54]. Moreover, glioma-associated oncogene homolog 1 (Gli1)+ pericyte loss was shown to induce capillary rarefaction and proximal tubular injury [55]. Of note, since pericytes have been previously linked and are closely related to MSCs [56], this also raises the question whether a subset of pericytes might be MSCs, and as such contribute to kidney regeneration. In fact, many studies have found a multipotent progenitor-like role for pericytes in various tissues [35,57,58].

Taken together, microvascular rarefaction directly contributes to the pool of myofibroblasts that are responsible for the excessive generation of ECM proteins that are the main constituent of scar tissue in fibrosis. In addition, pericyte-to-myofibroblast transition causes detachment of pericytes from the vascular wall, resulting in unstable capillaries that in itself would cause rarefaction [52]. Nevertheless, the main impact of rarefaction on the pathogenesis of chronic renal failure is caused by a loss in renal perfusion that further exacerbates medullary ischemia and drives the development of interstitial fibrosis, which is mediated by the augmented expression of TGF-β and connective tissue growth factor (CTGF) [59]. Thus, microvascular rarefaction may well function as a rate-limiting pro-fibrotic switch in the pathogenesis of chronic renal failure. Indeed, therapies targeting endothelial cell–pericyte interaction, e.g., aimed at PDGFR-β or VEGF receptor signalling, could prevent myofibroblast transition and limit the development of fibrosis [60,61,62], illustrating the key role of the capillary network in kidney injury and as potential therapeutic target.

Based on the above, it is clear that the complex vascular architecture of the kidney generates multiple perivascular compartments, each with their own specific functions and requirements. Therefore, future research focusing on an in-depth classification of renal pericytes by characterizing subpopulations based on their location, cell morphology, and function is required. As such, novel imaging modalities that aim to get access to small sized blood vessels non-invasively might provide this essential information. As exemplified by the field of neurobiology [12], the well-defined categorization of different subtypes of pericytes could provide new avenues for the development of targeted therapy for vascular malfunction.

## 4. Vascular Imaging Modalities

Different kidney diseases reflect a characteristic pattern of ultrastructural alterations. As a result of technological advances in the field of biomedical imaging, renal physiological and pathophysiological mechanisms were unraveled over the last few decades [63]. By focusing on anatomical and morphological changes of tissue architecture, our knowledge about renal disease enlarged progressively which improved diagnostics and provided innovative treatment opportunities. However, dynamical alteration of blood vessels has mainly been ignored due to the challenge to investigate vascular behavior in time-series experiments. As a result, there is an unmet medical need to develop non-invasive imaging techniques to monitor the hemodynamics of the renal microcirculation [16].

It would also be interesting to link imaging outcomes to (novel) biomarkers in the vascular nephrology field. For example, we demonstrated noncoding RNAs to be tightly linked to vascular injury [64,65]. Combining these measurements may yield novel (causal) relations and novel possibilities for diagnosis. Moreover, when novel imaging modalities are coupled with the recent development of single cell-based techniques such as single-cell RNA sequencing and spatial transcriptomics [53,66,67], this could allow an unprecedented in-depth analysis of the composition and dynamics of the renal vasculature. The following sections summarize already available ex vivo and in vivo imaging modalities for investigating morphological and functional aspects of the renal microvasculature.

### 4.1. Ex Vivo

A lot of our knowledge of the renal microvasculature is derived from comprehensive ex vivo analysis of tissue biopsies. Accordingly, cell-based therapy is often evaluated by tissue sectioning and staining. Even though there are many studies investigating the therapeutic effect of MSCs on the renal vasculature [68], few research groups have taken advantages of sophisticated imaging modalities, such as microcomputed tomography (micro-CT), to assess MSC therapy by examining the 3D architecture of the renal vasculature [69,70,71,72,73,74].

#### 4.1.1. Microcomputed Tomography (Micro-CT)

The introduction of microcomputed tomography (micro-CT) by Flannery et al. [75] in 1987 has opened new avenues for studying the intact vasculature of rodents in order to gain knowledge of disease mechanisms with a high spatial resolution. This ex vivo modality enabled the visualization of the renal microvascular architecture and the quantification of glomerular number, spatial distribution, and volume, which can be used as an indicator for the pathophysiological state of the whole organ [76]. The resolution in one 3D field of view with 10,243 voxels allowed the visualization of afferent and efferent arterioles, as well as the glomerular capillaries of rodent kidneys. In rats, a reconstituted voxel size of 21 μm was used [77] and in pigs the renal vasculature was studied with a voxel size of 40 μm and scan field of view of 1.2 mm [78]. Advances within the field of micro-CT provided the opportunity to image the nephron blood vessels of the rat with a voxel resolution of 1 μm within a scan field of view of 2 mm [79].

Based on the quantification technique developed by Hillman et al. [80] using conventional CT, vessel architecture and vascular volume within different renal tissue compartments were determined in line with similar studies, which evaluated renal vessels based on histological tissue sections [77]. Interestingly, by applying imaging modalities such as micro-CT, the importance was raised to investigate peritubular capillaries and their involvement in pathological conditions in addition to the conventional way of thinking about the role of the glomerular capillary network.

Early structural changes of the microvasculature can be visualized and detected by micro-CT, and therefore it is not surprising that several molecular mechanisms on the vascular level involved in kidney disease were identified by micro-CT. In various renal disease models, such as in polycystic kidney disease (PKD), a correlation was described between pathology and a decreased microvasculature as determined by micro-CT with a resolution of 17 μm voxel size [81]. Moreover, an increased cortical microvascular density was observed in hypercholesterolemia as an early sign of progressive renal morphological damage [78]. In rats with chronic bile duct ligation, cortical hypoperfusion was detected by micro-CT, which may explain the disturbance of salt and water retention with further disease progression [82]. Besides, in stenotic kidneys the increased oxidative stress was linked to renal microvascular remodeling and treatment possibilities were proposed through chronic antioxidant intervention [83].

A major advantage of micro-CT is that the axial, as well as the radial geometry of vascular systems can be defined [84]. Besides visualizing the renal vascular architecture in 3D, the spatial density of microvessels can be appreciated [78,83], vessel density and size can be determined with a diameter up to 80 μm in various anatomical renal compartments [77,78,83,85], and tortuosity of the vessels can be observed [83]. Moreover, the vascular capillary volume of glomeruli, as well the peritubular capillaries could be distinguished and quantified within the cortex [82].

A standardized quantification protocol has been widely used to investigate microvascular alteration within the well-defined cortical and medullary compartments on structural level in order to determine the vascular density and diameter [77,82]. Ngo et al. [84] performed a comparison study of micro-CT and light microscopy and concluded that the quantification of the renal vasculature geometry acquired by micro-CT is a feasible and accurate technique. The only added value of light microscopic imaging in comparison to micro-CT is that it permits distinguishing between arteries and veins through the possibility to visualize the vascular wall. However, by applying micro-CT, arteries of 100 and 60 μm in diameter of rats and rabbits, respectively, could be visualized in 3D. However, small-sized blood vessels smaller than 10 μm cannot be identified properly by micro-CT, urging the need to reverting back to immunohistochemistry in order to capture even the smallest renal capillaries [85].

#### 4.1.2. Light Sheet Fluorescence Microscopy (LSFM)

With the introduction of light sheet fluorescence microscopy (LSFM), high-resolution imaging of large volumes can nowadays be achieved in a reasonable amount of time [86,87]. Through the availability of LSFM, the interest was shifted from routinely applied conventional histological techniques that include sectioning the tissue, followed by staining the microvasculature, towards imaging the tissue in its whole. Volumetric analysis is favorable because not only a selected part of the tissue is examined, but also the dynamic character of vessel architecture and behavior is preserved in 3D view.

To keep the 3D information, a biological specimen is made transparent via various optical tissue clearing (OTC) protocols to minimize light scattering and light absorption for further fluorescent staining [87]. In the recent years, OTC methods gained on popularity since 3D imaging provides the opportunity to study intact organs were possible due to modern advances of LSFM. Construction of an intact organ or even a whole animal with a resolution on cellular level in 3D can be acquired within minutes [88]. With a comparable resolution to confocal fluorescence microscopy, LSFM however, has a two orders magnitude better signal-to-noise ratio, dramatically reduces fluorophore bleaching and phototoxic, enabling large-scale imaging processing required for OTC [89]. Additional advantages are that recorded number of frames and recording speed is greater, whereas the overall imaging duration is much shorter.

Ever since the introduction of LSFM, several OTC protocols were improved and refined for different tissue specimens and organs derived from several species. In the last years, the non-toxic solvent-based clearing by ethyl cinnamate (ECi) is widely applied to clear murine kidneys [88,90,91]. This protocol is less time consuming, as for example pioneering protocols CLARITY (clear lipid-exchanged acrylamide-hybridized rigid imaging), CUBIC (clear, unobstructed brain/body imaging cocktails and computational analysis), and/or DISCO (three-dimensional imaging of solvent-cleared organs), yet it offers a relatively reasonable clearing of the kidney with a low amount of autofluorescence left. Remarkable work was accomplished by Ertürk and colleagues [92], who successfully cleared an entire human kidney by a new tissue permeabilization approach called SHANEL (small-micelle-mediated human organ efficient clearing and labeling). The cortex zone was determined to have dimensions of around 2742 ± 665 mm (mean ± SD) that contained glomerular capillaries with a diameter of 221 ± 37 mm, and afferent arteriole had a diameter of 71 ± 28 mm. Moreover, a highly sophisticated deep learning-based framework for quantifying the neuronal vasculature after OTC, called VesSAP (vessel segmentation & analysis pipeline), has been developed within the field of neuroscience [93].

Despite the fact that a lot of great progress has been achieved in the field of OTC in recent years, some disadvantages are still remaining since the expression of endogenous fluorophores is mostly not satisfactorily preserved limiting the use of transgenic animals. However, a major concern is that the morphological size of the tissue and consequently of the vasculature is altered by the harsh solvents required for OTC. In addition, the tremendous amount of data produced by LSFM remains a challenge, not only for proper data storage and handling but also for quantitative analysis [87].

In a nutshell, one important advantage of applying techniques such as micro-CT or LSFM is that spatial vessel distribution can be captured and structural rarefaction of the vascular network is identified with a suitable resolution to image almost all of the renal capillary structures. However, both techniques require fixation and can therefore only be performed ex vivo. To monitor morphological and functional alterations of the renal microvasculature, in vivo time imaging strategies are desired.

### 4.2. In Vivo

The application of in vivo imaging modalities would offer the opportunity to evaluate cell-based therapy in real time and validate possible therapeutic effects on the vascular level. In consequence, advances in the field of in vivo biomedical imaging are pressing for studying MSC-based effects on the renal vasculature. Only a hand full of studies has applied in vivo imaging to examine MSC-action, utilizing multiphoton microscopy (MPM) [94], CT [95,96], and magnetic resonance imaging (MRI) [95,96,97].

#### 4.2.1. Multiphoton Microscopy (MPM)

Multiphoton microscopy (MPM) depends on the simultaneous absorption of two or more photons only within the focal plane, which became available in 1995 [98]. Dynamic processes can be visualized in vivo at the cellular level, and, besides studying the renal vascular blood flow [63], MPM offers the opportunity to monitor various renal microvascular segments in real time. The high resolution allows the visualization of the afferent and efferent arterioles and of glomerular capillaries. Even though it is technically possible to access the medullary microcirculation, it nevertheless remains a challenge due to the penetration depth [98]. However, it is feasible to visualize an entire glomerulus with an approximate diameter of 100 μm and get access to dynamic processes at the cellular level.

Importantly, in vivo microvascular leakage can be visualized and quantified through Evans blue extravasation in fibrotic kidneys by MPM [99]. The number of perfused capillaries was quantified and the diameter was determined far below 10 μm. Recently, our research group applied MPM to provide in vivo evidence that human pluripotent stem cell (hPSC)-derived kidney organoids formed a functional connection with the pre-existing renal vasculature in mice after renal subcapsular transplantation [100,101].

The advantages of MPM are the absence of out-of-focus fluorescence and a restricted photo bleaching within the focal region [98]. However, one of the disadvantages is the limited imaging depth that requires application of an abdominal imaging window for accessing the renal vasculature in vivo [102,103]. This abdominal window permits in vivo imaging for several weeks up to one month; however, the insertion of such an imaging window requires invasive surgery and is associated with chance of inflammation. Moreover, imaging windows are sometimes lost and tissue necrosis can occur [102]. Obviously, this imaging modality is not translatable into clinical practice.

An interesting alternative to monitor capillary blood flow non-invasively at the bedside is achievable since the introduction of hand-held vital microscopy (HVM) into clinical practice [104]. Even though this imaging modality is based on an entirely different technology, i.e., side stream and incident dark field video-microscopes, it offers real time assessment of superficial located capillaries. Novel clinical implemented algorithms, known as MicroTools software packages enable automated microvascular imaging analysis of total and perfused vessel density for accessing angiogenesis, vessel dilation/constriction, and fluid balance, as well as oxygen delivery capacity based on capillary hematocrit and velocity of erythrocytes [105]. However, to study the renal microvasculature, those modalities are not suitable due to their limited penetration depth.

#### 4.2.2. Computed Tomography (CT)

Non-invasive imaging modalities capable of monitoring and quantifying morphological and functional alterations of the renal microvasculature are highly demanded to determine the role of the microvasculature in disease progression to CKD. Even though a causal relationship between capillary rarefaction and progression of renal fibrosis has been recognized for many years, Ehling et al. [3] were the first to perform a non-invasive qualitative and quantitative analyses of anatomical and functional vascular alterations during the progression of CKD. A progressive decline of the renal blood volume has been observed by in vivo contrast-enhanced micro-CT in three murine models with progressive kidney fibrosis, i.e., ischemia-reperfusion injury (IRI), unilateral ureteral obstruction, and Alport mice. Besides functional changes of the microvessels, peritubular vascular loss correlated with the formation of fibrotic tissue within all three CKD mouse models. However, to gain information about branch points, vessel diameter and tortuosity, ex vivo micro-CT was required, indicating the need for emerging biomedical imaging technologies that provide access to microvessels in vivo with a close to cellular resolution.

A major advantage of CT is that the visualization of the renal vasculature is acquired within minutes with a reasonable resolution, providing 3D information about the vascular organization. Using iodine-based contrast agents the contrast is enhanced and an even more detailed depiction of the microvasculature is achieved. Recently, qualitative and quantitative assessment by micro-CT of murine kidneys in physiological and pathological conditions was refined by perfusing with phosphotungstic acid (PTA) to enhance the contrast within the blood vessels [106]. Even though the limitation that arteries and veins could not clearly be distinguished from each other, the resolution with a voxel size of 40 μm in vivo and a voxel size of 12.5 μm ex vivo captured the organization up to the level of arcuate blood vessels.

Another major disadvantage of using CT to monitor renal disease is, however, the necessity to utilize iodinated radiographic contrast agents. These contrast agents are known to cause nephrotoxicity, which is a contraindication for clinical application in patients with pre-existing renal impairment [107,108,109]. The acute impairment of the kidney due to the administrated contrast agent alters renal hemodynamics and causes medullary hypoxia which is in particular undesirable when investigating renal microvascular rarefaction. Similarly, gadolinium-based contrast agents widely utilized in MRI are eliminated by the kidney and seem to cause renal impairment [110].

#### 4.2.3. Magnetic Resonance Imaging (MRI)

MRI was introduced in clinical practice in 1980s and immediately become one of the most used imaging techniques [111]. MRI is a non-invasive and non-ionizing imaging modality that applies a strong magnetic field and by using T1 and T2 contrast agent’s alteration, the relaxation properties of blood can be detected. Moreover, magnetic resonance angiography (MRA) visualizes the vascular architecture of small animals by utilizing gadolinium-based contrast agent. However, a major disadvantage of MRA is the difficult usage of the required contrast agent. Fortunately, renal perfusion can be determined with and without the need of contrast agents offering both benefits and limitations [112].

Without the use of a contrast agent, spin-labeling takes advantages of endogenous water as a diffusible tracer that only makes it possible to quantify the perfusion within the renal cortex since the medullary transit time is too long to be captured. Moreover, to determine the renal blood flow, phase shifts of spins along one direction are measured, which implies the need of a perpendicular imaging plane towards the arteries of interest for achieving an accurate measurement [112]. Therefore, it is not surprising that this technique provides a big challenge when it comes to visualizing renal arteries, not to mention the small cortical capillaries.

Vessel functionality can be determined by quantifying the RBF by MR-perfusion and monitoring the oxygenation state by blood-oxygen-level dependent contrast (BOLD) imaging [112,113]. However, the main limitation of functional MRI is to achieve reliable and reproducible results in organs that are affected by respiratory movements, which include the kidney, even though it is less susceptible to movement artifacts in comparison to the liver or the bowel [112]. Despite the fact that 1.5 Tesla functional MRI-performed feasibility studies provided great promise when performing voxel-wise quantification [114], perfusion abnormalities could only be detected in pathological areas raising the question whether minor vascular alteration will be sufficiently detected. With the introduction of an MRI scanner with a magnetic field strength of 3.0 Tesla, the signal-to-noise ratio was tremendously improved [115,116], however the acquired resolution remains an issue.

The resolution provided by MRI is mainly depending on the magnetic field strength and can be optimized for any given magnetic field by adapting the pulse sequences [111]. The spatial resolution is mainly limited by the signal-to-noise ratio that requires a rapid acquisition time and generally achieving a resolution of 3×4 mm in pixel size [117]. Even when a high magnetic field of 7 Tesla is applied, the best resolution one can achieve with BOLD is around 500 μm. Besides the unsatisfying resolution and the limitation to visualize most dynamic processes, another major disadvantage is that MRI is linked to high costs and requires special facilities and maintenance.

#### 4.2.4. Ultrasound

Advances in ultrasound introduced a paradigm shift for non-invasive monitoring of structural and functional renal microvascular alteration and opened new avenues to explore small vessels with a portable system at a relatively low cost. In a rat model of acute ischemia caused by severe hypoperfusion for 1 h, renal blood flow was evaluated in real time by using color and pulsed-wave (PW) Doppler ultrasound [118]. Color Doppler imaging revealed also the difficulty to visualize arcuate arteries due to their relatively small size and the perpendicular probe positioning to capture the artery flow required for calculating blood velocity and applying Doppler angle correction. However, the blood velocity could only be calculated at the intra-renal arteries, i.e., segmental, interlobar, and arcuate arteries. Accordingly, information on the microcirculation could not be acquired due to the limitation of resolution provided by conventional ultrasound.

**Figure 3:**
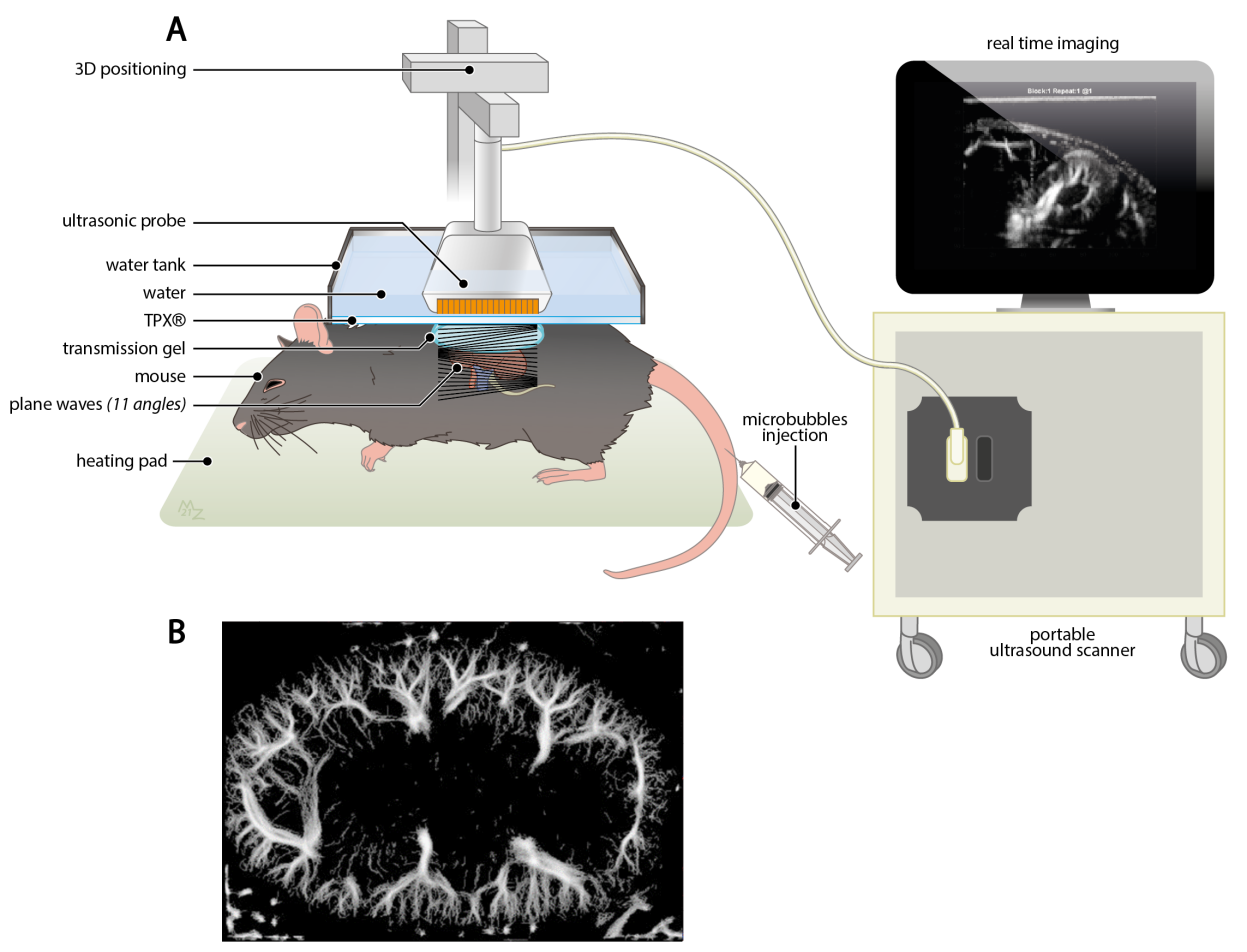
Ultrafast Doppler ultrasound imaging of the kidney is a highly promising technique to monitor renal microvascular rarefaction. (**A**) Schematic representation of experimental setup of ultrafast Doppler ultrasound imaging of the murine kidney. Portable ultrasound scanner is equipped with an ultrasonic probe that is operated through a 3D positioning motor system for real time imaging. A 3D printed water tank reduces motion artifacts and ensures acoustic impedance matching through water, TPX^®^, and transmission gel. To enhance the contrast, microbubbles are injected into the tail vein. (**B**) Representative image of renal ultrafast Doppler ultrasound imaging with injection of microbubbles to get access to capillary structures.

With the introduction of ultrafast Doppler ultrasound technology, a greater resolution could be achieved by unfocused wave transmission which is sending several synchronous waves at high frame rate simultaneously in one whole field of view, rather than scanning line-per-line through the application of focused beam transmission [119]. This plane wave transmission is the fundamental concept behind ultrafast Doppler ultrasound imaging and makes it possible to detect cortical vessels of a transplanted human kidney with a diameter below 1 mm [120]. Moreover, ultrafast Doppler ultrasound provides a highly favorable in vivo technique to monitor renal microvascular rarefaction in preclinical studies (Figure 3A). Despite the advances of ultrafast Doppler ultrasound, access on capillary level still relies on the utilization of contrast-enhanced agents [121]. Nevertheless, there are no worth mentionable safety concerns when utilizing contrast-enhanced agent for Doppler ultrasound, particularly in comparison to CT and MRI contrast agents that often show nephrotoxicity [122]. However, an incredible resolution of the murine renal vasculature can be accomplished in vivo (Figure 3B).

Ultrasound localization microscopy (ULM) solved the trade-off between spatial resolution and penetration depth by, on the one hand, applying ultrafast Doppler ultrasound imaging, and, on the other hand, making use of ultrasound contrast agents in form of gas-filled microbubbles [121,123,124]. Among others, Errico et al. [123] proposed ULM to image cranial microvessels with a diameter of 10 μm over the entire depth of the murine brain which is approximately 10 mm thick. In a recent publication, Demené et al. [125] could capture cerebrovascular blood flow dynamics at microscopic level deep in the human brain by tracking intravenously injected microbubbles individually to improve super-resolution imaging and enabling vascular resolution of up to 25 μm. To get a grasp on this amazing achievement, it is crucial to mention that no other non-invasive imaging modality could visualize the microvasculature in vivo below a millimetric scale. To reach this remarkable spatial resolution in vivo, there were two major challenges to overcome: the skull aberration and motion artifacts. Even though the application of ULM for abdominal organs such as the kidney is not hampered by the aberration of bone structures, the motion artifacts represent a great difficulty. However, recent in vivo studies successfully provided first attempts to image the renal vasculature by ULM [5,126,127].

Different vascular compartments within the rat kidney could be distinguished, and, by applying microbubbles, the resolution was increased to visualize the thin vessel bundles of the vasa recta that are separated by a distance of 400 μm from each other [5]. Additionally, axial blood velocity, i.e., below 2 mm/s, associated with the flow of renal microvessels has been estimated by tracking injected microbubbles with a diameter of 1 μm that can reach vessel diameter smaller than 20 μm. Song et al. [127] imaged the renal cortical microvessels of rabbits and could clearly separate vessels in vivo with a diameter of 76 μm. Although respiratory movement could be corrected, out-of-plane motion artifacts remain challenging and impossible to correct since imaging information could not completely be acquired [5].

Recently, AKI-to-CKD progression has been studied by contrast-enhanced ultrafast Doppler ultrasound in a mouse model of unilateral IRI [6]. By means of injecting microbubbles, 32 μm small renal blood vessels were identified and vascular changes in the kidney were quantified, i.e., renal blood volume, vascular density, and tortuosity. The vascular density of the cortex and corticomedullary junction acquired by ultrasound during in vivo imaging were in agreement with quantification obtained after CD31 immunostaining which is recognized as a golden standard in vascular biology. This is in line with another study, performed by Cao et al. [128], which illustrated that AKI severity can be determined by contrast-enhanced ultrasound by means of microbubble injection. Renal perfusion measurements in vivo closely correlated with renal injury determined on histological level. In accordance with those ultrasound studies, Hueper et al. [7] previously suggested that renal perfusion may predict AKI-to-CKD progression determined by MRI.

Microbubble-enhanced ultrasound imaging of the renal vasculature has already been successfully performed in humans to determine renal microvascular perfusion and showed great perspectives for diagnosis [129,130,131,132,133]. Interestingly, this imaging modality has been applied in renal transplantation to determine the perfusion status of kidney allografts which may provide a suitable non-invasive readout to predict acute rejection [134]. Besides operator dependency which may represent a limitation when utilizing ultrasound, the strength of inter-observer agreement was very high between two readers reflecting great feasibility when applying in clinical setting [132]. Due to the portability and the time-saving and simple customized application, ultrasound with contrast-enhanced microbubbles provides great perspectives for evaluating the renal microvasculature in clinical practice, especially in ICU patients [135]. This technology offers therefore great promise for the translation into clinical practice after successfully mastering correction of abdominal motion artifacts for robust microbubble tracking with a high precision.

## 5. Conclusions and Perspectives

The striking heterogeneity of the renal vascular architecture reflects its complex functional diversity and compartmentalization, with as logical consequence that studying microvascular alteration and rarefaction requires sophisticated imaging modalities. The development, application, and improvement of in vivo imaging modalities to study renal vascular diseases will provide a greater understanding of action of cell therapies such as MSC on the vascular level and may elucidate specific biomarkers that can be monitored during disease progression.

Besides qualitative assessment of the renal vascular damage evaluated in tissue sections, quantitative analysis of renal microvascular damage and capillary loss would pinpoint the specific sequence of event in functional and/or structural microvascular rarefaction. Future research should focus on developing cutting-edge imaging techniques to study the pathophysiological mechanism in time of the different vascular segments.

Advanced technologies of functional ultrafast ultrasound (fUS) and ultrasound localization microscopy (ULM) provide promising modalities for studying microvascular rarefaction and evaluating novel therapeutic approaches. Since Doppler ultrasound forms a non-invasive, portable, and safe modality for patients suffering from renal disease, it would allow monitoring renal microvascular changes over time with an adequate resolution. In regard to validating and refining cell-based therapy, it would provide a tremendous opportunity.

## Abbreviations

The following abbreviations are used in this manuscript:

Ang	Angiopoietin
AKI	Acute kidney injury
AP	Activator protein
ATF	Activating transcription factor
ATP	Adenosine triphosphate
AVR	Ascending vasa recta
BOLD	Blood-oxygen-level dependent contrast
CD	Cluster of differentiation
CKD	Chronic kidney disease
CLARITY	Clear lipid-exchanged acrylamide-hybridized rigid imaging
CT	Computed tomography
CTGF	Connective tissue growth factor
CUBIC	Clear, unobstructed brain/body imaging cocktails and computational analysis
DISCO	Three-dimensional imaging of solvent-cleared organs
DVR	Descending vasa recta
ECM	Extracellular matrix
ECi	Ethyl cinnamate
ESRD	End-stage renal disease
Fox	Forkhead box
fUS	Functional ultrafast ultrasound
GFR	Glomerular filtration rate
Gli	Glioma-associated oncogene homologue
HIF	Hypoxia-inducible factor
HVM	Hand-held vital microscopy
hPSC	human pluripotent stem cell
IRI	Ischemia-reperfusion injury
LSFM	Light sheet fluorescence microscopy
MBF	Medullary blood flow
micro-CT	Microcomputed tomography
MPM	Multiphoton microscopy
MRA	Magnetic resonance angiography
MRI	Magnetic resonance imaging
MSCs	Mesenchymal stromal cells
NG	Neuron-glial antigen
NO	Nitric oxide
OTC	Optical tissue clearing
PDGFR	Platelet-derived growth factor receptor
PKD	Polycystic kidney disease
PTA	Phosphotungstic acid
PW	Pulsed-wave
RBF	Renal blood flow
S1P	Sphingosine-1-phosphate
SCL	Stem cell leukemia
SD	Standard deviation
SHANEL	Small-micelle-mediated human organ efficient clearing and labeling
SMA	Smooth muscle actin
SMCs	Smooth muscle cells
TGF	Transforming growth factor
ULM	Ultrasound localization microscopy
VEGF	Vascular endothelial growth factor
VesSAP	Vessel segmentation & analysis pipeline
